# Structural Signature of Plasticity Unveiled by Nano-Scale Viscoelastic Contact in a Metallic Glass

**DOI:** 10.1038/srep29357

**Published:** 2016-07-07

**Authors:** Y. M. Lu, J. F. Zeng, S. Wang, B. A. Sun, Q. Wang, J. Lu, S. Gravier, J. J. Bladin, W. H. Wang, M. X. Pan, C. T. Liu, Y. Yang

**Affiliations:** 1 Institute of Physics, Chinese Academy of Sciences, Beijing, 100190, China; 2 Centre for Advanced Structural Materials, Department of Mechanical and Biomedical Engineering, City University of Hong Kong, Tat Chee Avenue, Kowloon Tong, Kowloon, Hong Kong SAR, China; 3 Université de Grenoble, CNRS, SIMAP Laboratory, UJF, Grenoble INP, BP46, 38402 Saint-Martin d’Hères, France

## Abstract

Room-temperature plasticity in metallic glasses (MGs) is commonly associated with local structural heterogeneity; however, direct observation of the subtle structural change caused by plasticity is vitally important but the data are extremely scarce. Based on dynamic atomic force microscopy (DAFM), here we show that plasticity-induced structural evolution in a Zr-Ni MG can be revealed via nano-scale viscoelastic contacts between an AFM tip and plastically deformed MG surface layers. Our experimental results clearly show a spatial amplification of the nano-scale structural heterogeneity caused by the distributed plastic flow, which can be linked to the limited growth, reorientation and agglomeration of some nano-scale energy-absorbing regions, which are reminiscent of the behavior of the defect-like regions with non-affine deformation as conceived in many theories and models. Furthermore, we are able to experimentally extract the thermodynamic properties of these nano-scale regions, which possess an energy barrier of 0.3–0.5 eV, about half of that for a typical shear transformation event that usually occurs at the onset of plasticity. The outcome of our current work sheds quantitative insights into the correlation between plasticity and structural heterogeneity in MGs.

Metallic glasses (MGs) are a promising structural material with excellent mechanical and physical properties^1^,^2^,^3^,^4^,^5^. However, although our understanding of the atomic structure in MGs has been greatly improved through the joint efforts from theories^6^, computational simulations^7^,^8^ and experiments^9^,^10^,^11^,^12^,^13^,^14^, the disordered atom packing in MGs lacking a long-range translational symmetry still defies the use of conventional means to explore their structure-property relations^15^. One fundamental reason is that MGs are thermodynamically metastable and, therefore, their atomic structure could evolve greatly under mechanical stress or temperature^16^,^17^,^18^,^19^,^20^. Consequently, it is difficult to trace back to any well-defined static structural features in the undisturbed state, like dislocations to crystalline metals, which enables the prediction of the physical/mechanical behavior of the MGs in the disturbed state. As a result, tremendous research efforts have been made over the past decades in the development of various theories^21^,^22^,^23^,^24^,^25^,^26^ and atomistic simulations^27^,^28^,^29^,^30^, which were aimed to elucidate the possible structural mechanisms that may give rise to the attractive mechanical properties of MGs. However, despite these enduring efforts, the structural origin of mechanical properties of MGs still remains intensely debated even today.

In principle, the amorphous structure of a glassy solid is inherited from the corresponding supercooled liquid after glass transition. Recent theories^31^,^32^,^33^ and atomistic simulations^34^ suggest that glass transition could be a critical-like phenomenon for fragile liquids, entailing the percolation of extremely slow regions with long relaxation times when the glass transition point is approached from above. Consequently, the structure of the glassy solids vitrified from fragile liquids tends to be heterogeneous in a dynamic sense, containing “liquid-like” regions with short relaxation times as embedded into “solid-like” regions with long relaxation times. This view of dynamic heterogeneity is validated for MGs with the recent results obtained from a variety of simulations^27^,^30^,^35^ and experiments^12^,^19^,^36^,^37^,^38^,^39^,^40^, which directly or indirectly indicate the presence of nano-sized “liquid-like” regions in MGs. These “liquid-like” regions are commonly related to the potential sites to accommodate local plasticity/relaxation as triggered by external thermo-mechanical agitations^19^,^20^,^27^,^39^,^41^.

To directly unveil the dynamic heterogeneity in MGs, it was first demonstrated by Liu *et al.*^40^ that dynamic atomic force microscopy (DAFM) with amplitude-modulation could be utilized as an effective tool. The idea behind this experimentation may be simply explained as follows. By taping the surface of a MG with a vibrating AFM tip, mechanical energies are transferred from the tip to MG surface layers. If the structure of a MG is homogenous and the tip-surface interaction remains elastic, the transferred energy should be stored as elastic strain energy and fully released upon tip retraction [Fig. 1(a)], thereby resulting in no structural contrast in the AFM phase image. However, if the structure of a MG is dynamically heterogeneous, a portion of the transferred energy would be dissipated away by the local “liquid-like” regions in a similar way as by a viscous liquid. As a result, viscoelasticity takes place as a manifestation of this local dynamic heterogeneity [Fig. 1(b)], hence giving rise to the structural contrast in the AFM phase images as shown by the recent experiments^12^,^38^,^40^. Although the above idea was commonly invoked for the interpretation of the AFM data, however, the correlation between the AFM phase image so obtained and the underlying dynamic heterogeneity in MGs is not one-to-one. As illustrated in Fig. 1(c,d), the tip-surface contact causes mechanical interactions between the underlying structure of the MG and the elastic field. If the interaction volume size is too large, it could obscure the structural heterogeneity of the MG. In other words, the AFM images depend on not only the structure of a MG but also the size of the interaction volume. In such a case, a micromechanical model is needed to relate the viscoelastic response of a MG under AFM tapping to its dynamic heterogeneity. In the current study, our goal is to use the DAFM method to probe the plasticity-induced structural evolution in MGs and develop a micromechanical model to understand the results in a more quantitative manner.

## Results

### Indentation of Thin-Film Metallic Glass with a Flat Punch

In the current study, a thin-film metallic glass (TFMG) with the composition of Zr_70_Ni_30_ was prepared by magnetron sputtering (see Method). The thin film has a thickness of 850 nm and was deposited onto a silicon wafer. The atomic structure of the as-deposited film was characterized by X-ray diffraction (see Supplementary Fig. 1) and height resolution transmission electron microscopy (HRTEM) (see Supplementary Fig. 2). Both experiments confirm the glassy state of the film. Here, we should stress that a thin film sample instead of a bulk is used because the DAFM technique is sensitive to surface roughness. Compared to a smooth thin film, fine polishing is usually required for bulk samples, which may damage the as-cast surface state and introduce surface defects. To reveal plasticity-induced structural evolution in MGs, indentation was conducted to introduce plastic deformation on the sample surface with a 10-μm diamond flat-end tip at a variety of indentation loads or displacements (see Method). Figure 2 shows the typical load-displacement curve obtained from the Zr_70_Ni_30_TFMG with a maximum indentation of 3 N. From the scanning electron microscopy (SEM) image (the insert of Fig. 2), the indentation mark can be clearly seen. To exclude the indentation edge effect, such as severe stress concentration near the edge of the indentation mark, DAFM experiments were conducted in the central part of the deformed region, as indicated by the red box in the SEM image, where plasticity is expected to occur in a non-localized manner from a macroscopic viewpoint. Here it should be noted no apparent shear bands were observed in the central regions of the indentation marks scanned. Moreover, the rough regions, which can also be seen from the SEM image, are excluded in our experiments and the scanned regions are mainly located in the deformed area with low roughness.

### Dynamic Atomic Force Microscopy Scanning

DAFM scanning was performed on the TFMG with an ultra-sharp diamond-like carbon coated (DLC) tip with a radius of ~1 nm before and after the indentation experiments (see Method). Surface topography and phase shift images were obtained simultaneously during the scanning, as shown in Fig. 3(a,b) respectively. According to our results, the measured root-mean-square (RMS) surface roughness is ~0.7 nm and the phase shift angles are all positive, indicative of repulsive tip-sample interactions due to a real material response^12^,^38^. According to the previous studies^12^,^38^,^40^,^42^,^43^, the phase shift angle 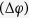 obtained from our AFM system is related to the energy (*E*_*di*s_) dissipated through viscoelasticity via 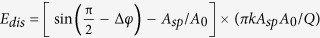, in which *A*_*s*p_ is the set-point amplitude; *A*_*0*_ is the free amplitude without tip-sample interaction and *k* and *Q* are the spring constant and quality factor of the AFM cantilever, respectively. Here it should be noted that the phase shift angle 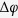 is not only related to the energy dissipation but also weakly dependent on the surface topography of the scanned area. Therefore, before using the above equation to compute the energy dissipation, the surface topography effect was already removed based on the method detailed in ref. 12 (Please also see Supplementary Note 1). Figure 3(c) shows the energy dissipation image obtained from an as-deposited TFMG. Evidently, the dissipated energies fluctuate on the nanometer scale and it can be proved that this nano-scale fluctuation in the local energy dissipation image has no correlation with the surface topography image (see Supplementary Note 1 and Note 2), hence indicative of a viscoelastic response from an intrinsically heterogeneous glassy structure. As discussed in the prior works^12^,^38^,^40^, the low-dissipation regions (**LDR**) may correspond to the regions rich in “solid-like” structures while the high-dissipation regions (**HDR**) to the regions rich in “liquid-like” structures.

Figures 4(a–c) display the typical energy dissipation images obtained at the indentation loads = 0 N, 40 mN and 3 N respectively. Evidently, plasticity causes an overall increase in the viscoelastic energy dissipation. Furthermore, as compared to an initial random homogeneous distribution of the HDR [Fig. 4(a)], some kind of plasticity-induced structural polarization, as manifested by the coalescence of the HDR, can be noticed at the indentation load of 40 mN [Fig. 4(b)]. With further increasing the indentation load to 3 N, the structural polarization becomes more significant with the HDR localized into the patterns resembling a quadrupolar symmetry as shown by the inset of Fig. 4(c). These quadrupolar-like patterns exhibit a preferred orientation close to ~45^0^ relative to the in-plane principal stress direction, which indicates that the observed structural polarization or localization is mainly driven by the maximum shear stress. For a further analysis, we performed 2D Fast Fourier Transform (FFT) of the AFM images [Fig. 4(a–c)]. As shown in Fig. 4(d–f), the spherical (ring-like) symmetry in the FFT of Fig. 4(a) confirms that the amorphous structure of the TFMG in the as-deposited state is isotropic with a random orientation of the individual structural features, such as HDR or LDR, in the real space^44^. In contrast, the FFTs [Fig. 4(e,f)] obtained after the occurrence of plasticity exhibit the preferred orientations, indicating plasticity-induced structural anisotropy^45^ as consistent with the AFM images [Fig. 4(a–c)]. In addition, it can be also noticed that the larger is the indentation load or the deeper is the indentation depth, the higher is the inclination angle exhibited by the FFT patterns, implicative of a higher degree of structural anisotropy. Additionally, this unique structural heterogeneity unveiled by DAFM is further confirmed by the scanning results of single-crystal silicon, which, in contrast, exhibits low energy dissipation value with extremely sharp distribution, as shown in Supplementary Fig. 5.

### Plasticity-Induced Structural Evolution

To further understand the plasticity-induced structural evolution in the TFMG, we performed a thorough statistical and geometrical analysis of the AFM images obtained. Figure 5(a) shows the typical distribution of the viscoelastic energy dissipation before and after the occurrence of plasticity. For each of the energy distribution, numerous sites in the central region of the corresponding indentation mark were scanned and consistent results were obtained (see Supplementary Fig. 6). As seen in Fig. 5(a), the overlap of the energy distributions in the low dissipation tail indicates that, on a statistic average, the LDR can be viewed as being almost “intact” during the plastic flow; however, the energy distribution obtained after the occurrence of plasticity becomes broadened and skewed to the high energy tail. This indicates that plasticity not only causes structural polarization as seen in Fig. 4(b,c) but also produces more HDR. Furthermore, by following the method in ref. 12 and taking the HDR as those above a cut-off value selected on the energy dissipation distribution, such as the top 5%, we also characterized the shape and orientation of these HDR by assuming their shape to be elliptical (see Supplementary Fig. 7). Figure 5(b) displays the ratio of the average long axis *D*_*max*_ to the short axis *D*_*min*_ measured for these HDR at different glassy states. Note that the magnitude of *D*_*max*_ or *D*_*min*_ may depend on the cut-off value; however, the ratio of *D*_*max*_*/D*_*min*_ is independent of the cut-off over a wide range of values as discussed in ref. 12. Interestingly, we observe that the ratio of *D*_*max*_*/D*_*min*_ increases from ~1.9 to ~2.4 or the shape of these HDR becomes slender with plasticity. In principle, this trend of shape change implies a higher stress concentration when the HDR are activated. Additionally, the average equivalent radius of these HDR, by assuming the shape of HDR to be a circular, increases from ~3 nm to ~6 nm with increasing applied stress, which confirms the growth of the HDRs.

### Viscoelastic Correlation Length

**Figure 1:**
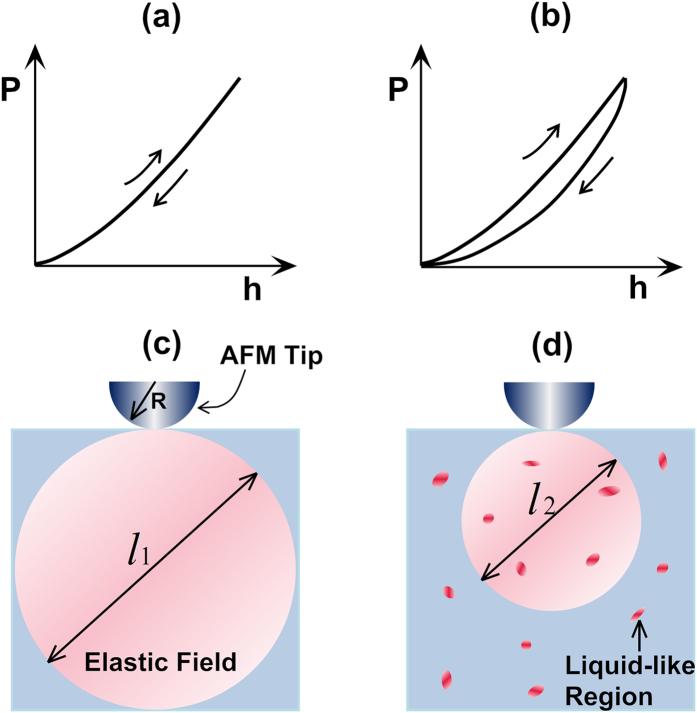
The schematic illustrations of the mechanical response for elastic and viscoelastic materials under AFM tip tapping. The typical load-displacement (*P-h*) curve for (**a**) elastic and (**b**) viscoelastic material; (**c**) the elastic interaction volume within an elastic material with its size *l*_*1*_ correlated with the elastic properties and AFM tip radius; and (**d**) the elastic interaction volume within a viscoelastic material with its size *l*_*2*_ correlated with the viscoelastic properties and AFM tip radius. Note that, if all conditions remain identical except the presence of liquid-like regions, *l*_*2*_ should be smaller than *l*_*1*_ because of the energy dissipation.

**Figure 2:**
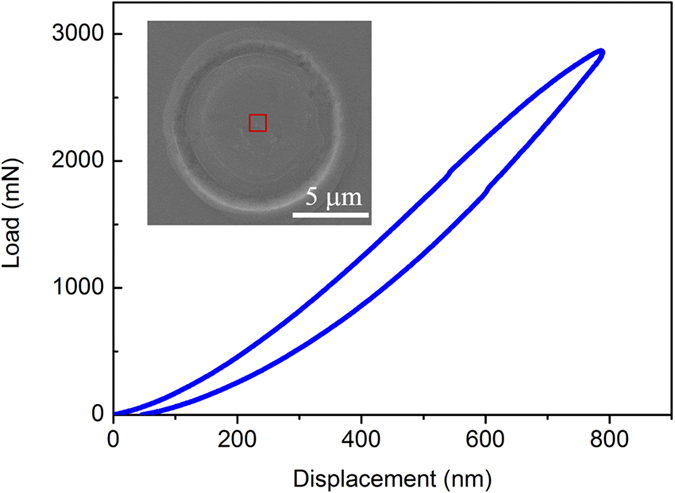
Load-Displacement curve in indentation experiments. The curve is obtained with a flat-end tip with radius of 5 μm and peak load of 3 N. The insert is the SEM image of the indentation area and the central part is indicated by the red box.

**Figure 3:**
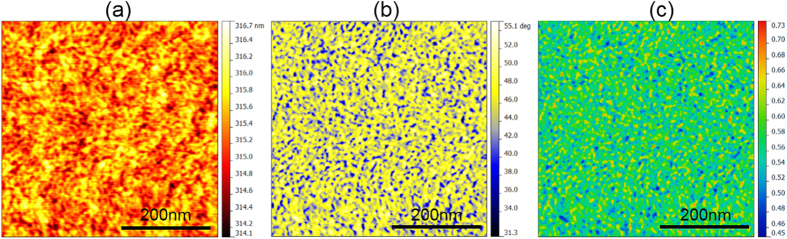
Typical AFM images obtained from the as-deposited Zr_70_Ni_30_ TFMG with a 1-nm DLC-tip. (**a**) The topography (height) image, (**b**) the phase image and (**c**) the energy-dissipation image derived from (**b**).

**Figure 4:**
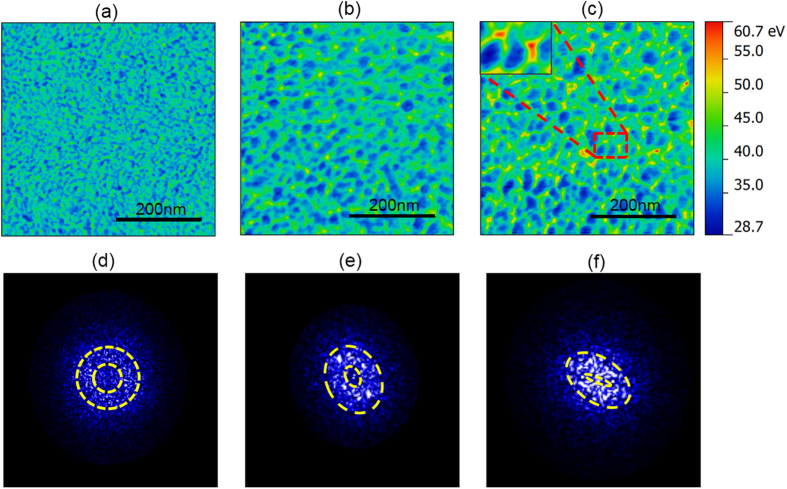
Energy dissipation images of Zr_70_Ni_30_ TFMG and the corresponding 2D FFT patterns. (**a–c**) Are energy dissipation images obtained at the as-deposited state, at the indentation area with the load of 40 mN and 3 N, respectively. The zoom-in image of the area boxed in red in (**c**) clearly shows the quadrupolar characteristics of HDR. (**d**–**f**) Are the corresponding FFT patterns of each image. The intensity distribution in the reciprocal space is indicated by the yellow circles.

**Figure 5:**
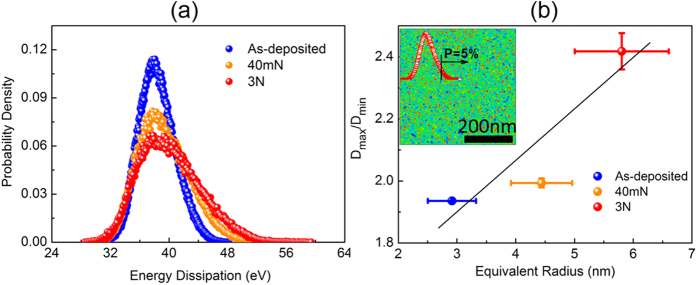
The energy dissipation spectra of Zr_70_Ni_30_ TFMG and characteristics of the HDR in Zr_70_Ni_30_ TFMG. (**a**) The spectra are obtained at the as-deposited state and at the indentation area with the load of 40 mN and 3 N. For each state, ten different sites of a 500 nm × 500 nm size were scanned. (**b**) The averaged aspect ratio versus the equivalent disk radius of HDR in Zr_70_Ni_30_ TFMG. Insets: normalized energy dissipation image (shown in Fig. 1(c)) and the corresponding energy spectrum (red curve). The top 5% of the total energy is defined as the HDR, which is highlighted in red in the image. *D*_*max*_ and *D*_*min*_ are the averaged maximum and minimum bounding lengths of the elliptical HDR, respectively. *R*_*eq*_ is the average size (radius) of these HDR by assuming that they take on a circular shape. The black line is drawn for eye guides.

**Figure 6:**
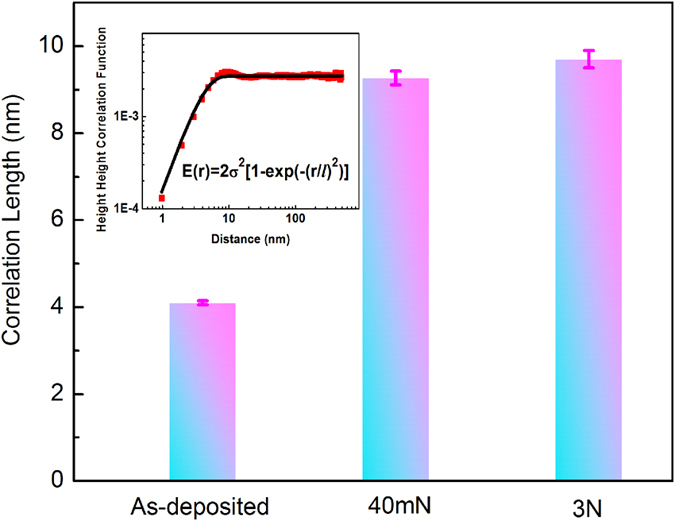
The correlation length of Zr_70_Ni_30_ TFMG. The correlation lengths of the energy dissipation images at different states. Inset: Estimation of correlation length by using equation 
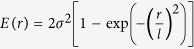
, in which *l* is the correlation length, *σ* is the root mean square energy dissipation.

Next, we carried out an in-depth analysis of the correlation length (*l*), which, as aforementioned, can be directly linked to the viscoelastic heterogeneity in the TFMGs (Fig. 1). In this work, the correlation lengths were evaluated from the energy dissipation image via the height-height correlation function 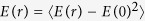, in which *E*(*r*) and *E*(*0*) denotes respectively the energy dissipation values at the coordinate (*x*, *y*) and the reference position (*x*_*0*_*, y*_*0*_). As shown by the inset of Fig. 6, the height-height correlation function of the energy dissipation images can be well fitted by a stretched exponential, from which the correlation length can be obtained. From Fig. 6, one can clearly see a dramatic increase in the correlation length from ~4 nm in the as-deposited film to ~10 nm after plastic deformation occurred. From the preceding discussions, one can easily envision that the correlation length is a measure of the tip-sample interaction (Fig. 1), which is strongly affected by both the tip size and the structural heterogeneity in the TFMGs. Since the tip radius used in the current study is about 1 nm and much smaller than the correlation length, the tip size effect can be ruled out. Furthermore, we also confirm that the vibrational amplitude of the AFM tip, which directly affects the applied tip repulsive force, also plays a negligible role in the measurement of the correlation length (see Supplementary Fig. 8). Therefore, we conclude that the change in the extracted correlation length is a reflection of a real structural change. In such a case, based on the aforementioned mechanism of viscoelastic dissipation, one may envision that the correlation length, which is one characteristic of the interaction volume between the AFM tip and the TFMG, should decrease when more HDR are activated. This is, however, just opposite to our experimental observations. Such a discrepancy indicates that the correlation length is not only affected by the size and number of the HDR as discussed in the previous work^12^,^38^,^40^ but also by some other factors that have not been explored before.

## Discussion

Based on the obtained experimental results, now we are at the position to discuss the possible physical mechanisms underlying the phenomenon of plasticity-induced structural evolution in the TFMG. To this end, we turn to a physical model early developed by Hentschel *et al.*^46^. This model relates the energy shared per particle to the size and dimensionality of a strained amorphous system during a plasticity event, which is Δ*U* = 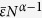, in which 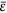 denotes the activation energy of the basic relaxation unit, *N* the number of particles in the system and *α*(=0.4) is a constant^47^,^48^. In line with these early work^46^,^47^,^48^, we can thus define the boundary of the interaction volume, as illustrated in Fig. 1, as corresponding to 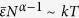, where *k* is the Boltzmann’s constant and *T* the experimental temperature. Consequently, a length scale (*l*) can be derived as follows:


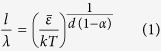


where *λ* is the mean distance between two basic relaxation unit and *d* is the dimension of the space in which the basic relaxation units interact. Note that in this model, the relaxation unit, which is the basic unit of HDR and responsible for energy dissipation in DAFM, is different but can be associated with the shear transformation zone (STZ) proposed for lower temperature plasticity mechanism^22^. In principle, the local regions containing more relaxation units, indicative of more HDR, are more responsive to applied stress and hence more flexible to willingly rearrange themselves, as a result, these regions are more prone to form STZ units. To compute the interaction length scale *l*, here we take *λ* ~ 1 nm, the order of magnitude for the size of a typical “flow unit”^49^,^50^,^51^,^52^,^53^. Table 1 lists the values of the correlation length *l* as a function of 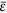 for *T* = 298 K and *d* = 2 or 3. Evidently, for the as-deposited TFMG, the computed *l* agrees very well with the experimental value for *d* = 3 and 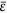 ~ 0.3–0.5 *eV*. Interestingly, within the same range of 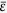, the computed *l* also matches the correlation length (~10 nm) obtained from the plastically deformed TFMG if *d* = 2. Given the fact that a similar viscoelastic relaxation process is activated by the vibratory tip in our AFM experiments and thus a similar activation energy is expected, this observation has a very important implication: i.e. the HDRs interact in a 3-dimensional space or in a homogeneous manner in the as-deposited TFMG while in a 2-dimensional space in the plastically deformed TFMG. In other words, although it cannot be directly observed on the TFMG surface, the dramatic increase in the correlation length hints the localization of the HDR into individual planes underneath the surface.

To verify the above scenario, the activation energy 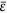 of the Zr_70_Ni_30_ TFMG is measured through the cyclic spherical indentation method devised in ref. 54, as detailed in the Supplementary Fig. 9 and Supplementary Table 1. At the equivalent testing frequency of ~1 kHz, the value of 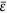 was measured to be nearly the same (~0.62 *eV*) in different glassy states. Considering the huge difference between the nanoindentation frequency (~1 kHz) and AFM frequency (~100 kHz) we estimate that the activation energy for the viscoelastic relaxation event in the AFM test should be ~0.5 *eV* according to the Arrhenius relation between the activation frequency and activation energy for a localized relaxation process, 
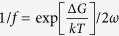
, where *ω* is the Debye frequency. This is consistent very well with the activation energy (0.3–0.5 eV) just inferred by comparing the experimentally obtained correlation length (4.1–9.7 nm) with the theoretical calculations (5.2–11.87 nm). Before proceeding to summary, it is worth mentioning that the correlation length, which is one characteristic of the interaction volume between the AFM tip and the TFMG, is affected by the size, density and geometrical characteristics of the high-dissipation regions (liquid-like structures) in MGs. Therefore, in practice, one may take it as a reflection of structural heterogeneity to rank different MGs. However, we should emphasize that the correlation length extracted in this study mainly originates from the surface layer (~4–10 nm) with a limited volumetric contribution, which may differ from the structural heterogeneity deduced for bulk ones^55^,^56^,^57^.

In summary, the plasticity-induced structural evolution in the Zr_70_Ni_30_ TFMG, which is otherwise hidden in conventional mechanical tests, is revealed in this work through the dynamic AFM technique which functions as a result of the viscoelastic heterogeneity intrinsic to the TFMG. Our experiments and analyses clearly show that plasticity causes the proliferation, size change and localization of the individual HDR. By comparison, in the sense of statistic averaging, the LDR remain almost unaltered by the plastic flow. The high resistance of the LDR to plasticity and the ease propensity of the HDR to undergo morphological change constitute the prominent structural features of plasticity-induced structural evolution in the TFMG. Since dynamic heterogeneity is intrinsic to glassy solids^58^,^59^, we envision that the methodology developed in this work should be applicable to study other glassy solids in the important engineering problems, such as creep, fatigue and fracture, where the structural understanding is usually a prerequisite for improving the structural properties of the glasses.

## Method

### Sample preparation

The TFMG was deposited from a Zr–Ni target by means of reactive DC magnetron sputtering using an Alliance Concept AC450 device. The substrate was silicon (100), positioned at a distance of 70 mm from the target. The vacuum limit was 5 × 10^−7^ mbar and the argon working pressure was 3 × 10^−3^ mbar. The delivery power for deposition was 300 W.

### Plastic deformation

Indentation experiments with peak loads of 40 mN and 3 N were performed on the TFMG by using a flat-end tip with radius of 5 μm. The angle between the indentation area and the horizontal plane is less than 0.1^0^. To calculate this angle, we first define three points (not in a straight line) in the indentation area and then find their coordinate (*x*_*i*_*, y*_*i*_*, z*_*i*_) through the quick approach process. With knowing these three points, we can define the indentation plane. As for the horizontal plane, the value of the *z*_*i*_ is the same for the three points. Once these two planes are well-defined, we can calculate the angel between them. Meanwhile, the amorphous structure of the deformed TFMG was also confirmed by HRTEM (See Supplementary Fig. 10).

### AM-AFM experiment

The AM-AFM experiment was employed under a proportional-integral feedback control on a Vecco^TM^ multimode AFM platform. To achieve ultrahigh spatial resolution, a diamond-like carbon coated AFM tip with radius of ~1 nm was used, which possesses a spring constant *k* of ~40 N/m and a damping factor of ~700. In order to obtain the real structural contrast, we perform a series of DAFM experiments by scanning a same area on the surface of the Zr_70_Ni_30_ TFMG with different *A*_*sp*_/*A*_*0*_ ratios^38^, in which *A*_*0*_is the vibration amplitude of the tip before if contacts the sample surface and *A*_*sp*_ is the set-point amplitude. Based on the obtained height and phase images, we find that the tip-surface interaction is attractive at a high ratio (>0.75) and repulsive at a low ratio (<0.75). Thus, to keep the repulsive tip-sample interaction, a relatively low *A*_*sp*_/*A*_*0*_ ratio (*A*_*sp*_/*A*_*0*_ = 0.15, *A*_*0*_ is ~20 nm and *A*_*sp*_ is ~3 nm) was utilized.

## Additional Information

**How to cite this article**: Lu, Y. M. *et al.* Structural Signature of Plasticity Unveiled by Nano-Scale Viscoelastic Contact in a Metallic Glass. *Sci. Rep.* **6**, 29357; doi: 10.1038/srep29357 (2016).

## Supplementary Material

Supplementary Information

## Figures and Tables

**Table 1:** The computed correlation length (*l*) as a function of the activation energy for *T* = 298 K and *d* = 2 or 3 for the Zr_70_Ni_30_ TFMG based on the equation of 
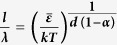
 in comparison with the experimental value.

	Activation energy (*eV*)	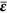 = 0.3	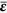 = 0.4	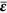 = 0.5	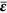 = 0.6	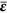 = 0.7	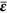 = 0.8	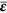 = 0.9	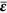 = 1.0
Theory	Length scale (*d* = 2)	7.76	9.86	11.87	13.82	15.71	17.56	19.37	21.15
	Length scale (*d* = 3)	3.92	4.60	5.20	5.76	6.27	6.76	7.21	7.65
Experiment	Length scale (As-deposited TFMG)	4.10 ± 0.05							
	Length scale (Deformed TFMG *P* = 40mN)	9.27 ± 0.16							
	Length scale (Deformed TFMG *P* = 3N)	9.70 ± 0.20							

The unit of the correlation length is nanometer.
