# Supplementary material for: Structural Signature of Plasticity Unveiled by Nano-Scale Viscoelastic Contact in a Metallic Glass
Source: Sci Rep. 2016 Jul 7;6:29357. doi: 10.1038/srep29357 (PMC4935946; doi:10.1038/srep29357)
Supplement: Supplementary Information [file srep29357-s1.pdf]

Supplementary Information for "Structural Signature of Plasticity Unveiled by Nano-Scale Viscoelastic Contact in a Metallic Glass"

Y. M. Lu[1,2,†], J.F. Zeng[1,†], S.Wang[1], B.A.Sun[1], Q.Wang[1], J.Lu[1], S.Gravier[3], J.J.Bladin[3], W.H.Wang[2], M.X.Pan[2,*],C.T.Liu[1] and Y.Yang[1,*]

[1]Centre for Advanced Structural Materials, Department of Mechanical and Biomedical Engineering, City University of Hong Kong, Tat Chee Avenue, Kowloon Tong, Kowloon, Hong Kong SAR, China

[2]Institute of Physics, Chinese Academy of Sciences, Beijing 100190, China

[3]Université de Grenoble, CNRS, SIMAP Laboratory, UJF, Grenoble INP, BP46, 38402 Saint-Martin d'Hères, France

Correspondence and requests for materials should be addressed to Y. Y. (yonyang@cityu.edu.hk) and M.X. P. (panmx@aphy.iphy.ac.cn).

Supplemental materials including

Figs. 1-10

Table 1

Text 1-2

References

Supplementary Figures

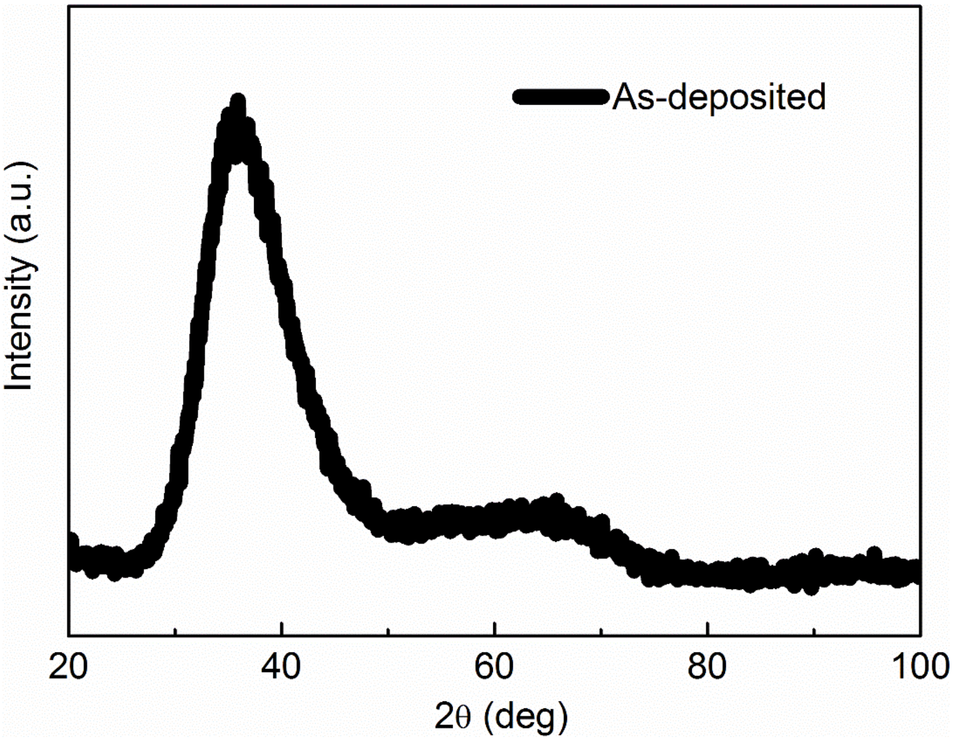

Supplementary Figure 1. X-ray diffraction patterns of $Zr_{70}Ni_{30}$ TFMG at the as-deposited state.

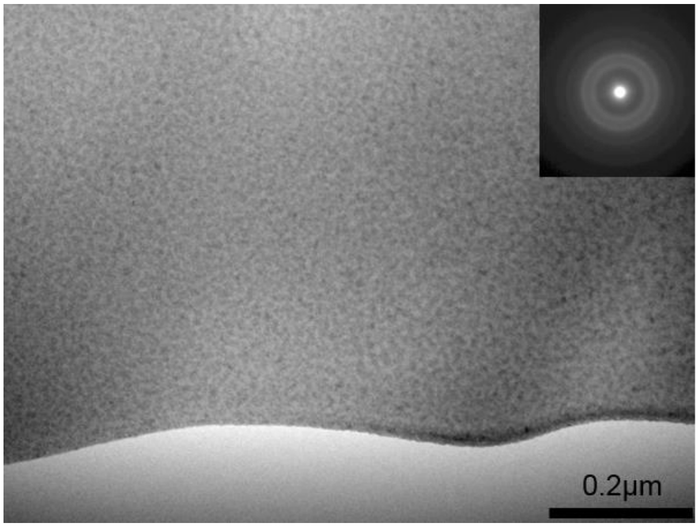

Supplementary Figure 2. HRTEM image and SAED pattern of the $Zr_{70}Ni_{30}$ TFMG at the as-deposited state.

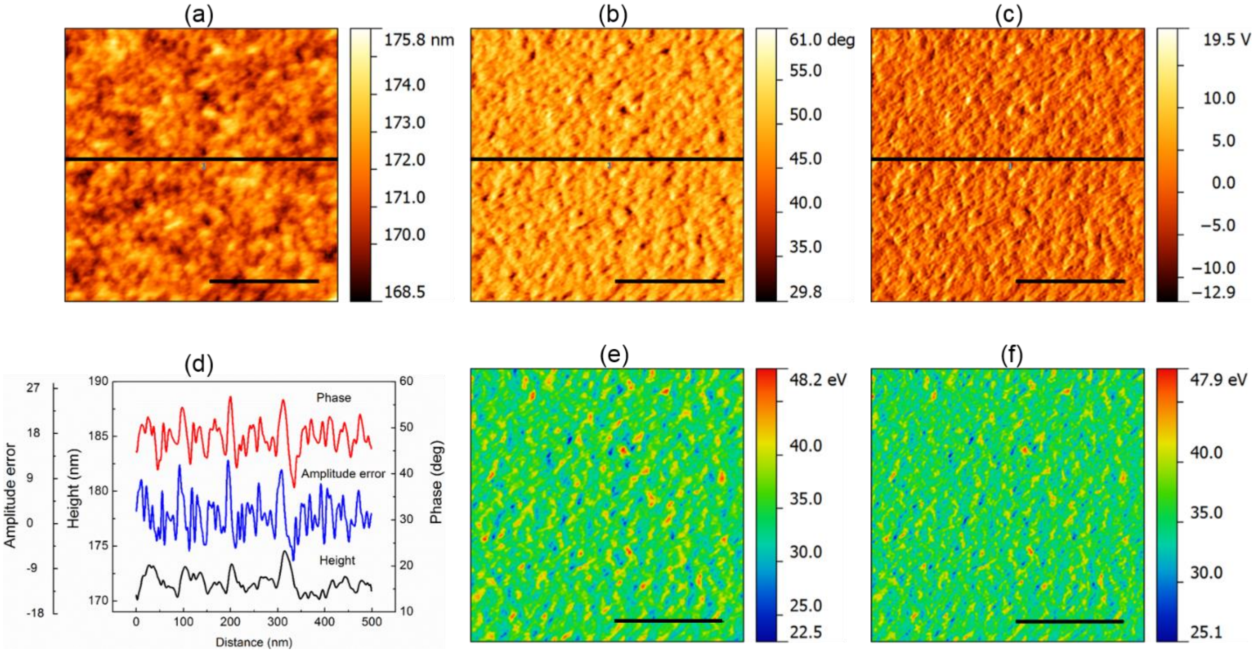

Supplementary Figure 3. AFM images and energy dissipation images of the $Zr_{70}Ni_{30}$ TFMG obtained at the as-deposited state. (a) the height image, (b) the phase image, (c) the amplitude-error image (d) line-scan results obtained at the middle section (black line), energy dissipation images obtained (e) before and (f) after deconvolution of the surface topography from the phase image. The scale bars are 200nm.

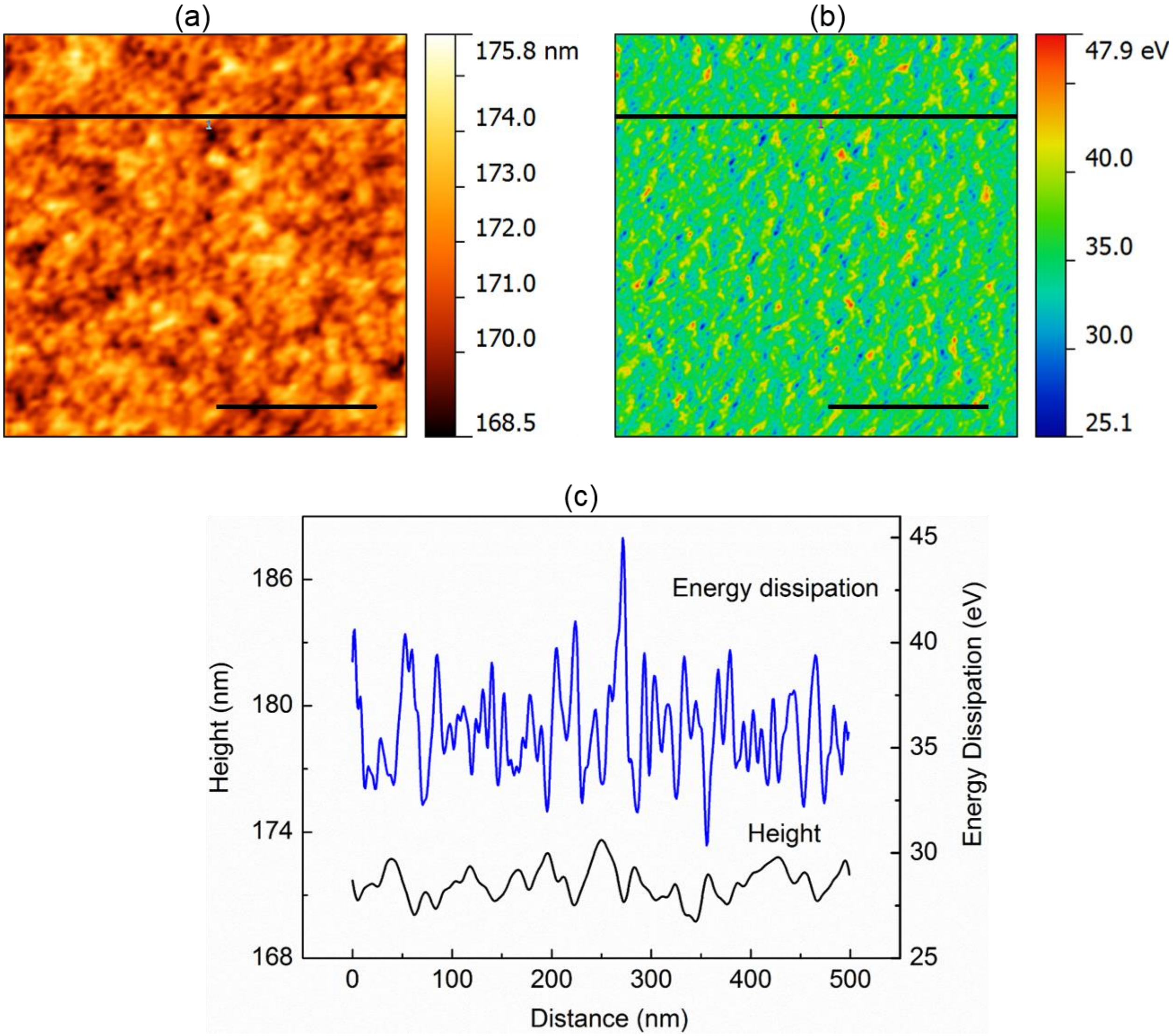

Supplementary Figure 4. (a) The height image, (b) energy dissipation image (after deconvolution) and (c) line-scan results of the black line indicated in (a) and (b) of the $Zr_{70}Ni_{30}$ TFMG obtained at the as-deposited state.

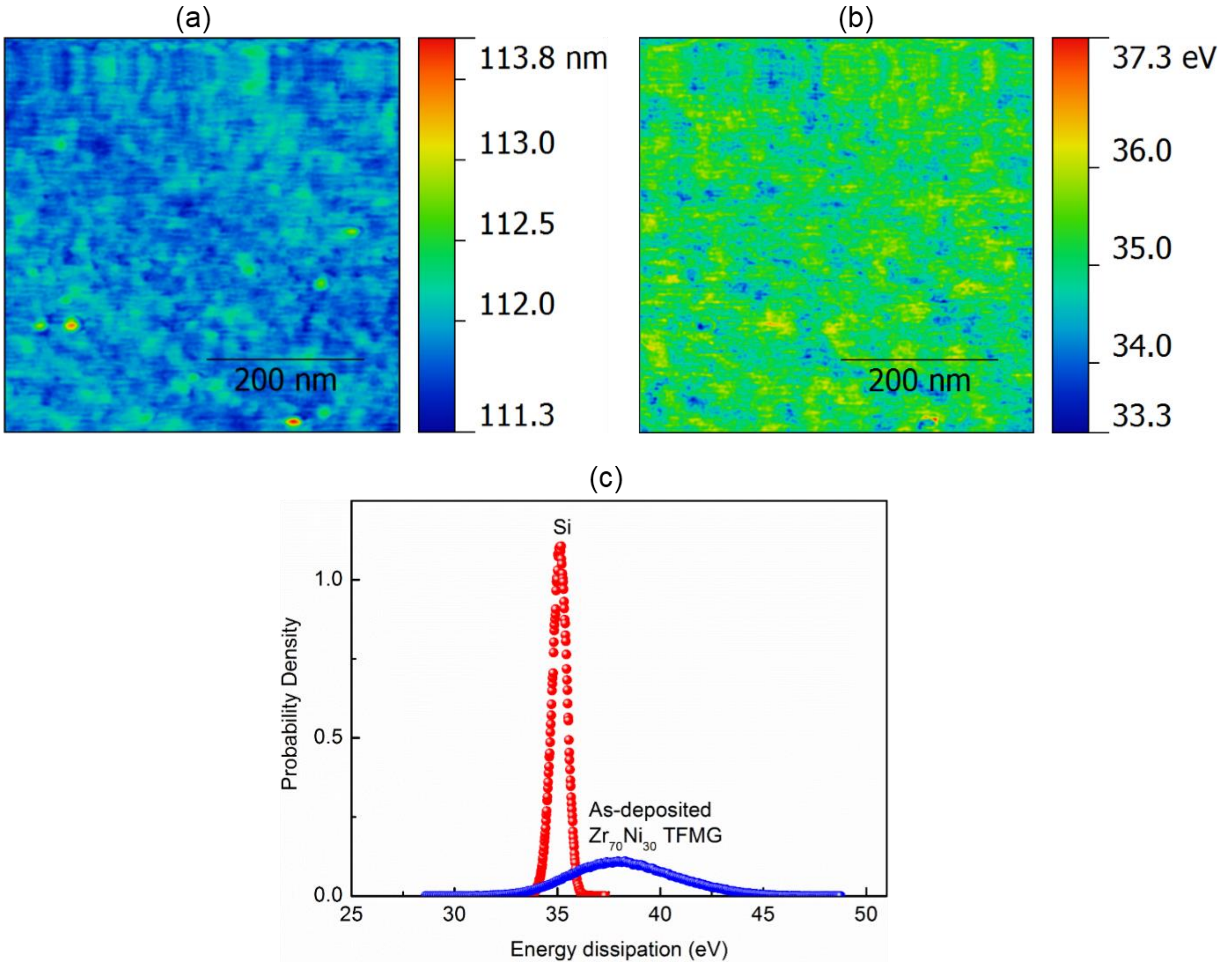

Supplementary Figure 5. (a) The height image, (b) energy dissipation image (after deconvolution) and (c) energy dissipation spectra of single-crystal silicon and $Zr_{70}Ni_{30}$ TFMG obtained in DAFM experiments.

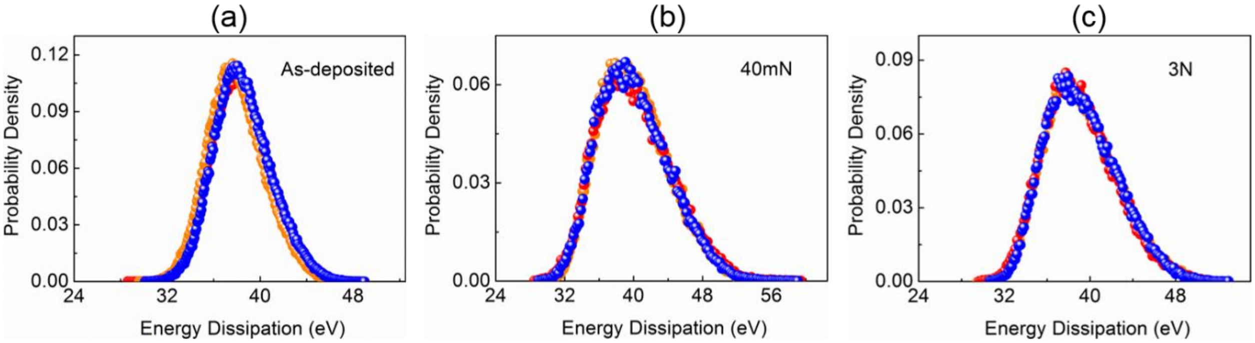

Supplementary Figure 6. The energy dissipation spectra of $Zr_{70}Ni_{30}$ TFMG obtained at (a) the as-deposited state and at the indentation area with the load of (b) 40mN and (c) 3N. For each state, the data obtained at three different sites of a 500 nm × 500 nm size were shown.

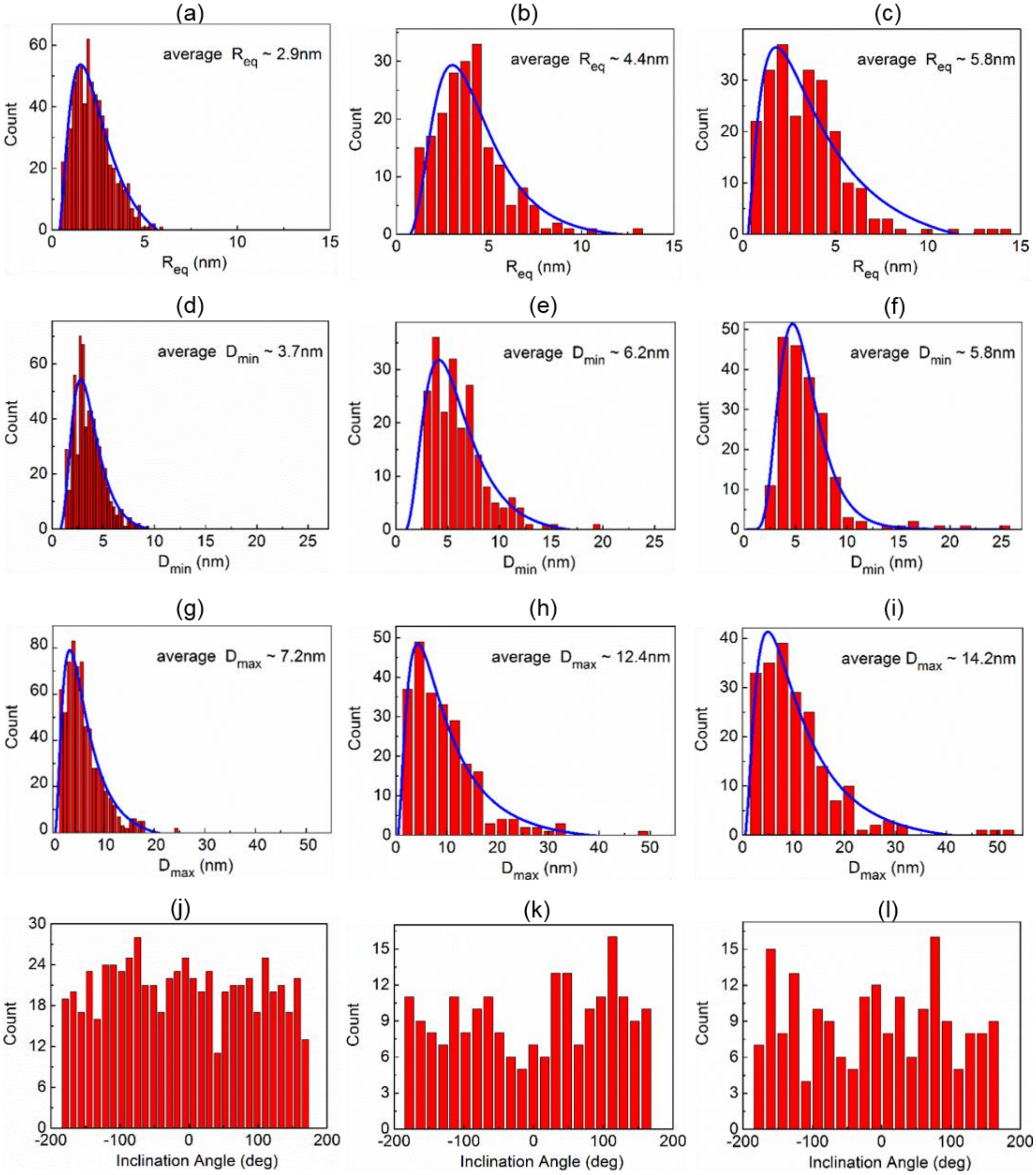

Supplementary Figure 7. Different geometric parameters of the elliptical HDR in $Zr_{70}Ni_{30}$ TFMG. (a)-(c) are histograms of the average size (radius), R_{eq}, by assuming their shape to be circle, (d)-(f) the lengths of the short axis, D_{min}, (g)-(i) the lengths of the long axis, D_{max} and (j)-(l) inclination angle of the HDR at the as-deposited state and at indentation areas with the load of 40mN and 3N. The blue curves are fitted results of the data by using the lognormal function.

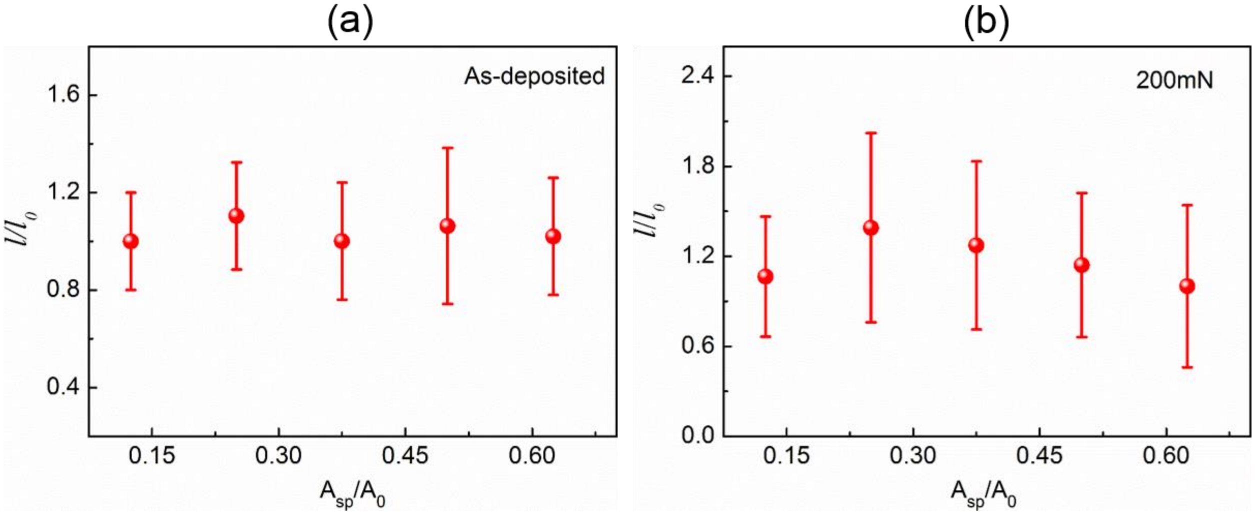

Supplementary Figure 8. The correlation length versus the ratio of A_{sp}/A_0 for $Zr_{70}Ni_{30}$ TFMG obtained at (a) the as-deposited state and (b) the indentation area with the load of 200mN. The correlation length is scaled by the average correlation length l_0.

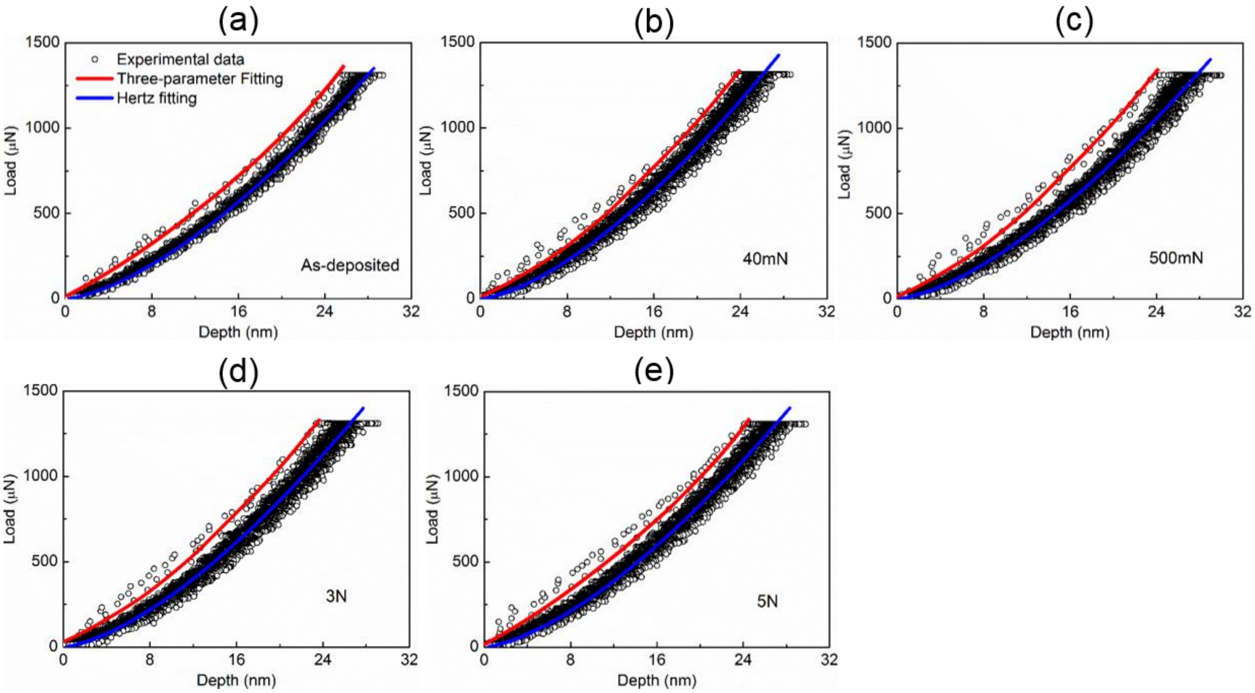

Supplementary Figure 9. The experimental and calculated curves for $Zr_{70}Ni_{30}$ TFMG in nanoindentation experiments obtained at (a) the as-deposited state and the indentation areas with the load of (b) 40mN, (c) 500mN, (d) 3N and (e) 5N. The calculated loading curves (red) are obtained by using the three-parameter viscoelastic model and the unloading calculated curves (blue) are obtained by using Hertzian Theory. The loading, holding and unloading time in nanoindentation experiments is 0.002s, 0.2s and 0.2s, respectively.

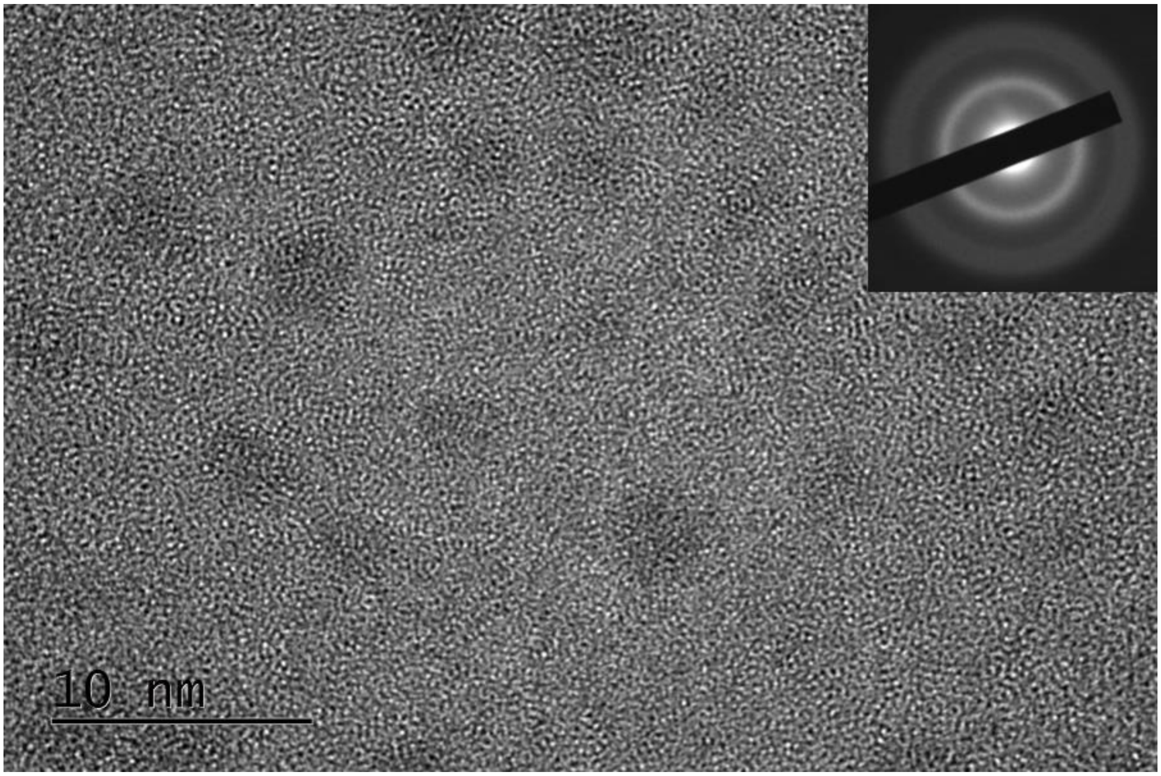

Supplementary Figure 10. HRTEM image and SAED pattern of the $Zr_{70}Ni_{30}$ TFMG at the indentation load of 5N.

Supplementary Tables

Supplementary Table 1. The calculated parameters in three-parameter viscoelastic model for $Zr_{70}Ni_{30}$ TFMG at different states.

	As-deposited	40mN	500mN	3N	5N
G_I (*GPa*)	28.2±1.6	28.1±2.0	27.5±1.7	27.7±1.6	26.8±1.0
G_{II} (*GPa*)	9.1±0.3	9.8±0.2	10.2±0.4	11.0±0.2	11.9±0.2
η ($MPa \cdot s$)	5.6±0.3	5.4±0.9	6.8±0.8	7.6±0.2	8.2±0.2
Activation energy (*eV*)	0.61±0.01	0.61±0.02	0.61±0.01	0.62±0.01	0.62±0.01

Supplementary Notes

Supplementary Note 1

In AM-AFM scanning, the tip contacts the sample surface intermittently and the real-time phase shifts arising from tip-sample interaction were recorded point by point. According to previous studies[1], two factors contribute to the recorded phase shift in DAFM: topography effect and energy dissipated from the bulk, that is, $\Delta\varphi = \Delta\varphi_{surf} + \Delta\varphi_{bulk}$. Decomposing the phase image into moments of bulk effect allows us to detect the structural characteristics of the sample surface. In DAFM, $\Delta\varphi = \beta\delta A_{sp}$, where δA_{sp} is the recorded amplitude error and β can be calculated by minimizing the standard deviation of $\Delta\varphi - \beta\delta A_{sp}$. Thus, $\beta = \frac{E(\Delta\varphi \cdot \delta A_{sp}) - E(\Delta\varphi) \cdot E(\delta A_{sp})}{D^2(\delta A_{sp})}$, where $E(\cdot)$ and $D(\cdot)$ represent the mean and standard deviation of a physical quantity.

Supplementary Figs. 3(a)-(c) show the height, phase and amplitude-error images obtained simultaneously in DAFM experiments at the as-deposited state. We can see that the phase and amplitude-error images exhibit similar spatial patterns while the height and phase images don't look alike. This observation can be further justified through the line-scan results shown in Supplementary Fig. 3(d). Thus, it is clear that the topography and the bulk effect both contribute to the measured phase shift. In the following analysis, the surface effect is removed from the phase image by using the equation $\Delta\varphi - \beta\delta A_{sp}$ based on the computed value of β. For comparison, Supplementary Fig. 3(e)-(f) display the energy dissipation images converted from Supplementary Fig. 3(b) before and after deconvolution. It is evident that the surface effect is negligible on energy dissipation. That's because the β value deduced from the original phase and amplitude-error images is small (0.5) due to the smoothness of the thin film. In our paper, all energy dissipation images are converted from the corresponding phase images in which the surface effect is removed.

Supplementary Note 2

In principle, when AFM tip contacts the sample surface, energies can be dissipated via either attractive or repulsive force between the tip and sample surface[2]. Based on previous studies[3,4], repulsive forces produce a real material response and can be used to characterize structural features while attractive forces, such as Van der Waals force and capillary force, produce artifacts and mislead the structural analysis. In our AFM system, a repulsive force corresponds to a positive phase shift while an attractive force to a negative phase shift[4]. Thus, to obtain a real structural contrast, we have to obtain positive phase shift in the whole phase image. In AFM, the tip-sample force can be controlled by adjusting the mean distance between the tip and sample surface and a repulsive force is favored at a short distance[2]. Therefore, a repulsive force can be obtained by adjusting the ratio of A_{sp}/A_0 since this ratio determines the average tip-sample distance. In our studies[3,4], a series of experiments with different A_{sp}/A_0 values were performed and the results show that positive phase shift can only be obtained at relatively low ratios (A_{sp}/A_0 <0.75). To ensure a repulsive tip-sample interaction, all DAFM experiments were conducted under the same ratio of 0.15. In this regard, it is believed that the energy dissipation images, which is converted from the corresponding positive phase shift images, reveals the real structural contrasts and have no correlation with the surface topography. Moreover, from the line-scan results in Supplementary Fig. 4(c), it can be further confirmed that the energy dissipation image after deconvolution is indeed uncorrelated with the height image.

References

1 Stark, M., Moller, C., Muller, D. J. & Guckenberger, R. From images to interactions: High-resolution phase imaging in tapping-mode atomic force microscopy. *Biophys. J.* **80**, 3009-3018, (2001).

2 Garcia, R. & San Paulo, A. Attractive and repulsive tip-sample interaction regimes in tapping-mode atomic force microscopy. *Phys. Rev. B* **60**, 4961-4967, (1999).

3 Yang, Y. *et al.* Fractal growth of the dense-packing phase in annealed metallic glass imaged by high-resolution atomic force microscopy. *Acta Mater.* **60**, 5260-5272, (2012).

4 Zeng, J. F. *et al.* On the use of atomic force microscopy for structural mapping of metallic-glass thin films. *Intermetallics* **44**, 121-127, (2014).
